# Influence of the SARS-CoV-2 pandemic and infection on musculoskeletal function

**DOI:** 10.1038/s41598-025-17780-x

**Published:** 2025-09-12

**Authors:** Alonja Reiter, Alena Haack, Elina Larissa Petersen, Stefan Blankenberg, Karl-Heinz Frosch, Darius Thiesen, Johannes Keller

**Affiliations:** 1 Department of Trauma and Orthopeadic Surgery, University Medical Center Hamburg-Eppendorf, Hamburg, Germany; 2 Department of Cardiology, University Heart and Vascular Center Hamburg, University Medical Center Hamburg-Eppendorf, Hamburg, Germany; 3 Center for Population Health Innovation (POINT), University Heart and Vascular Center Hamburg, University Medical Center Hamburg-Eppendorf, Hamburg, Germany; 4 German Center for Cardiovascular Research (DZHK), Partner Site Hamburg/Kiel/Lübeck, Hamburg, Germany; 5 Department of Trauma Surgery, Orthopeadics and Sports Traumatology, BG Hospital Hamburg, Hamburg, Germany

**Keywords:** SARS-CoV-2, Physical activity, Muscle quality, Muscle quantity, Public health, Risk factors

## Abstract

As of September 2023, the number of SARS-CoV-2 infections worldwide has exceeded 770 million, with ongoing research recognizing its long-term health effects. This study investigates the effects of the pandemic on participants with and without SARS-CoV-2 infection on musculoskeletal status. The Hamburg City Health Study (HCHS) is an ongoing, prospective, population-based cohort study (trial registration number: NCT03934957). Participants (n = 2660) examined prior to the SARS-CoV-2 pandemic were compared with non-infected participants (n = 2660) in a 1:1 matched cohort analysis after the pandemic onset. Furthermore, participants who had recovered from a SARS-CoV-2 infection (n = 428) were compared with non-infected participants (n = 1712) in 1:4 matched cohort analysis. Increased relative fat mass and reduced skeletal muscle mass were observed in the post-pandemic group. No significant differences were observed between the groups regarding handgrip strength and timed up and go test. The comparison of the infection group with non-infected participants showed no significant differences in relative fat mass, handgrip strength and timed up and go test. The skeletal muscle mass was increased in the infection group but linear regression analysis adjusted for body surface area revealed no significant differences. This prospective study shows no association between SARS-CoV-2 infection and changes in basic musculoskeletal parameters in the cohort studied, suggesting that the infection may not be a significant cause of major deterioration in musculoskeletal function. Instead, it appears that lifestyle changes in daily routines and physical activities associated with the pandemic itself are the main influence on the musculoskeletal system.

**Supplementary Information:**

The online version contains supplementary material available at 10.1038/s41598-025-17780-x.

## Introduction

By September 2023, the global count of confirmed SARS-CoV-2 infections have exceeded 770 million^1^. A growing body of research has demonstrated the multi-organ involvement of SARS-CoV-2 during its acute phase^2^. Furthermore, multiple investigations have explored its long-term effects, such as Long-Covid and persistent repercussions on pulmonary, cardiac, and renal functions, thromboembolism, and neurological complications^3–5^. However, the impact of the SARS-CoV-2 pandemic itself and an infection on the musculoskeletal system remains unclear.

Progressive and generalized loss of muscle mass and function represents a significant public health concern, particularly in the older population^6^. Earlier studies have suggested a potential link between SARS-CoV-2 infection and an increased risk of musculoskeletal decline. The infection triggers a cytokine storm, leading to muscle catabolism. This heightened inflammation can lead to muscle catabolism, where muscle proteins break down faster than they are synthesized, resulting in muscle loss^7–10^. This concern is accentuated by lockdowns and the pandemic’s influence on physical activity levels, potentially contributing to an elevated prevalence of musculoskeletal impairment, especially among older adults already vulnerable to muscle loss^11^. Although information available about the link between SARS-CoV-2 infection and declining musculoskeletal health remains limited, studies indicate that an infection may lead to persistent impairments in muscle mass and function in certain individuals. However, the existing studies are restricted by small sample sizes and retrospective designs. Moreover, most studies have focused on the impact on pre-existing musculoskeletal dysfunction on the course of a SARS-CoV-2 infection, rather than the influence of SARS-CoV-2 on the development of muscle-related impairments^12–14^. The underlying mechanisms of post-SARS-CoV-2 musculoskeletal impairment remain unclear, though factors such as prolonged hospitalization, bed rest, systemic inflammation, cytokine storm, and the exacerbation of pre-existing comorbidities (e.g., diabetes, cardiovascular disease) may contribute to its development^15,16^. In the present study, we thus hypothesized that SARS-CoV-2 infection is associated with indices of impaired musculoskeletal health, thereby increasing the risk of functional and structural muscle loss, particularly in individuals with symptomatic infection.

The aim of this study was to investigate the influence of the SARS-CoV-2 pandemic itself and delineate the influence of SARS-CoV-2 infections on musculoskeletal health.

## Materials and methods

### Study population

The Hamburg City Health Study (HCHS) is an ongoing, prospective, population-based cohort study at the University Medical Center Hamburg-Eppendorf (UKE), Germany, to gain knowledge and deep phenotyping about major chronic diseases. The recruitment started in February 2016 and includes a random sample of 45–74-year-old Hamburg residents drawn from the residents’ registration office. The study was registered at ClinicalTrials.gov (NCT03934957)^17^.

**Fig. 1:**
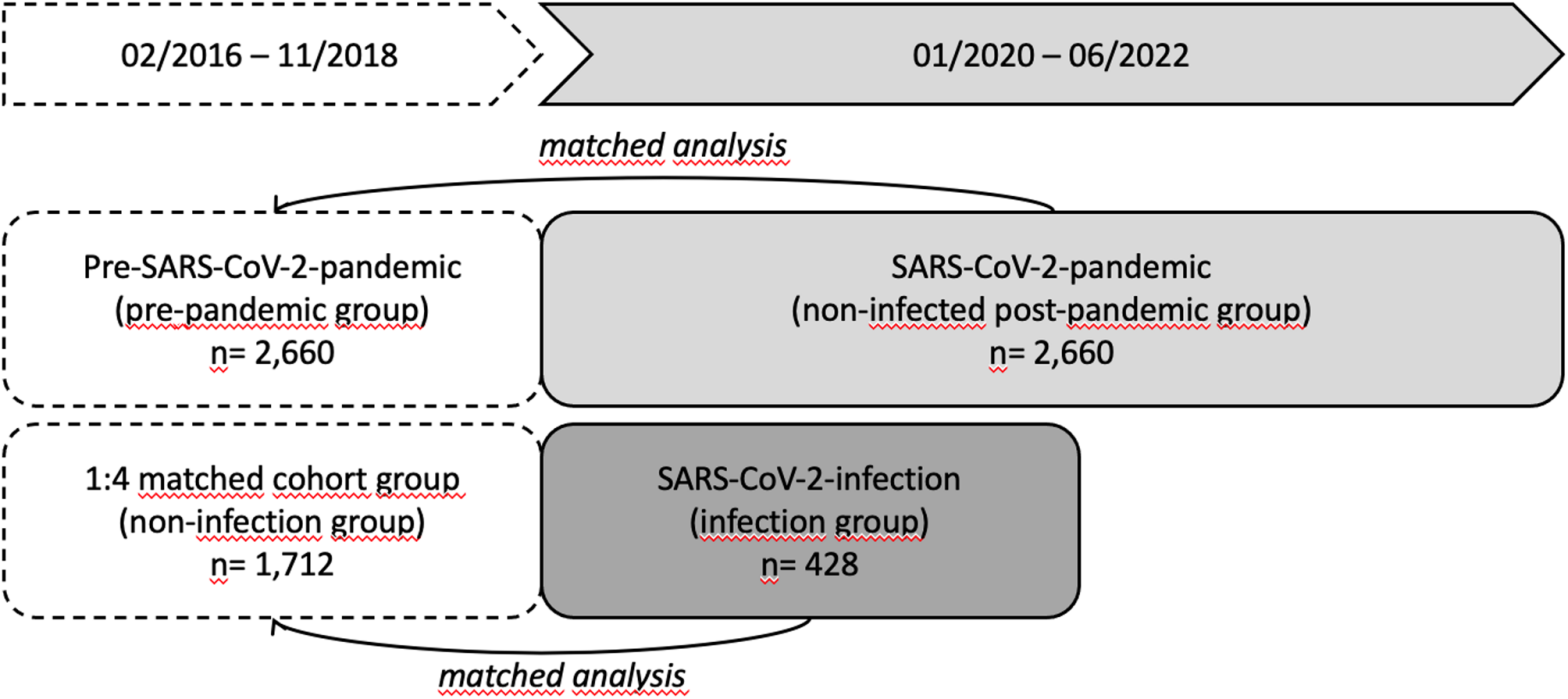
Detailed timeline representing the process of participant recruitment and examination undertaken within the Hamburg City Health Study, highlighting the specific groups that were systematically matched and analyzed in comparison.

To investigate the influence of a SARS-CoV-2 infection, the analyses included participants of the additionally started post-SARS-CoV-2 program of the HCHS. These participants were recruited via a call in the newspaper or by invitation after confirmed SARS-CoV-2 infection within the UKE and underwent the standard program of the HCHS. All participants defined as post-SARS-CoV-2 had a confirmed SARS-CoV-2 infection, verified by a positive polymerase chain reaction (PCR) test for SARS-CoV-2, at least four months before study participation, between March and December 2020, and were between 45 and 74 years of age at the time of recruitment. All participants signed written informed consent forms. The local ethics committee, the State of Hamburg Chamber of Medical Practitioners (PV5131), approved the inclusion of post-SARS-CoV-2 individuals and the study’s extension of the HCHS, ensuring compliance with the Declaration of Helsinki. The severity of the disease was based on self-reported symptoms at the time of SARS-CoV-2 infection and retrospectively documented into four categories: asymptomatic, mild symptoms, moderate symptoms not requiring hospitalization, and moderate symptoms necessitating hospitalization. A detailed study description has been published elsewhere^5^. In addition, non-infected participants from the ongoing HCHS, which enrolled on the study between January 2020 and June 2022 (i.e., also during the pandemic), to investigate the association between SARS-CoV-2 pandemic and musculoskeletal impairment were selected. Matched controls were chosen from the first 10,000 HCHS participants who enrolled between February 2016 and November 2018 to exclude the possibility that they were infected with or vaccinated against SARS-CoV-2 (Fig. 1). Before matching, all participants with missing values in all four outcomes (hand grip strength right/left, skeletal muscle mass and timed up and go) were excluded.

### Outcomes

All participants underwent the same study protocol of the HCHS. The examination was performed by trained study nurses at one single time point^17^. Hand grip strength was measured by a hand dynamometer (Jamar Plus Dynamometer), a specialized device designed for this purpose. The participants were instructed to hold the dynamometer with one hand and apply maximal force by squeezing as hard as possible. This procedure was repeated three times for each hand to obtain a reliable average grip strength value. Participants’ mobility, balance, and gait were measured by the timed up and go test. It involves measuring the time it took for them to rise from a chair, walk a distance of three meters, turn around, return to the chair, and sit down again. While various techniques have been described to assess skeletal muscle mass in humans^18^, in our study this parameter was assessed using Bioelectrical Impedance Analysis (Medical Composition Analyzer seca 515/514). Small electrical currents were passed through the body via four electrodes, one on each hand and each foot. The impedance data obtained was also used to estimate relative fat mass.

To identify patients with sarcopenia, the definition of sarcopenia provided by the European Working Group on Sarcopenia in Older People (EWGSOP) in 2018 was used. This definition necessitates meeting at least two out of three criteria: low muscle strength, indicated by grip strength falling below 27.0 kg for men and 16.0 kg for women; low muscle quantity, characterized by reduced skeletal muscle mass below 20.0 kg for men and 15.0 kg for women or skeletal muscle mass below 7.0 kg/m^2^ for men and 5.5 kg/m^2^ for women identified through either Magnetic resonance imaging (MRI), computed tomography (CT), Dual-energy X-ray absorptiometry (DXA) or Bioelectrical Impedance Analysis (BIA); and low physical performance, identified through a timed up and go test taking 20 s or more^19^.

### Statistical analyses

The matching of controls was performed with the k- nearest neighbor matching on the propensity score by age at baseline examination (± 2 years), sex (male and female) and education (three categories according to the International Standard Classification of Education) in a 1:4 (subjects after SARS-CoV-2 infection versus matched controls) and 1:1 (subjects since beginning of SARS-CoV-2 pandemic versus matched controls) case and control ratio, respectively. Comparisons between groups were performed using the Kruskal–Wallis test for continuous variables and the Chi-square test for categorical variables. Continuous variables are described as the median and interquartile range (IQR), and categorical variables as frequency and percentage.

The association between outcomes and SARS-CoV-2 infection (respectively SARS-CoV-2 pandemic) was analyzed using multiple linear mixed models with the matching cluster as a random intercept. All analyses were additionally adjusted for body surface area. An additional sensitivity analysis was performed with further adjustment for residual differences in comorbidities. In a further sensitivity analysis, we included physical activity as a covariate. *p*-values were adjusted for multiple testing with Bonferroni correction for eight comparisons. All analyses were performed in R version 4.1.0.

## Results

### Impact of SARS-CoV-2 pandemic on musculoskeletal parameters

This matched cohort analysis included a total of 5320 participants, with 2660 participants who were examined before the SARS-CoV-2 pandemic (pre-pandemic group) and 2660 patients who were examined during the SARS-CoV-2 pandemic (post-pandemic group). The baseline characteristics, including sociodemography, and anthropometry, were found to be comparable in both groups. There were no significant differences between the pre-pandemic and post-pandemic groups in terms of age, BMI, height, weight, and body surface area.

However, several parameters indicative of musculoskeletal health showed statistically significant differences between the groups. The mean relative fat mass was 29.10% (IQR: 24.20–34.50) in the pre-pandemic group and 31.30% (IQR: 25.60–37.20) in the post-pandemic group. The relative fat mass (*p* < 0.001) showed statistically significant differences between the two groups, with increased relative fat mass observed in the post-pandemic group. In terms of comorbidities, there were statistically significant differences between the groups. Hypertension (*p* = 0.002) and cancer (*p* = 0.002) were significantly more frequent in the pre-pandemic group, while chronic lung disease e.g. asthma, COPD (*p* = 0.002) was more common in the post-pandemic group. In relation to the questionnaire assessing physical activity, there was a significant difference in the average hours of physical activity per week. The questionnaire included all types of sports activities. Pre-pandemic participants reported an average of 3 h per week, while post-pandemic participants reported 2.5 h per week (*p* = 0.001). For detailed baseline characteristics, please refer to Table 1.

**Table 1:** Baseline and functional characteristics: pandemic cohort vs. matched controls.

	Pre-SARS-CoV-2 pandemic (matched controls) (n = 2660)	Post-SARS-CoV-2 pandemic (pandemic cohort) (n = 2660)	*p*-value
Demographics			
Age, years	54.00 [50.00, 59.00]	54.00 [50.00, 59.00]	0.551
Sex, female	1216 (45.7)	1220 (45.9)	0.934
Education^a^			0.769
Low	52 (2.0)	55 (2.1)	
Medium	1142 (42.9)	1117 (42.0)	
High	1466 (55.1)	1488 (55.9)	0.551
Anthropometry			
BMI, kg/m^2^	25.75 [23.34, 28.89]	25.76 [23.20, 29.09]	0.764
Height, cm	173.70 [166.90, 180.70]	173.80 [166.80, 181.00]	0.318
Weight, kg	78.80 [68.00, 89.75]	79.30 [68.30, 91.10]	0.319
Body surface area (DuBois)	1.94 [1.78, 2.09]	1.94 [1.78, 2.11]	0.259
Relative fat mass, %	29.10 [24.20, 34.50]	31.30 [25.60, 37.20]	< 0.001
Lifestyle			
Status of employment			0.031
Full-time employed	1548 (60.6)	1608 (61.5)	
Part-time employed	442 (17.3)	491 (18.8)	
Marginal employed	108 (4.2)	109 (4.2)	
Not employed	165 (6.5)	119 (4.6)	
Pension	293 (11.5)	286 (10.9)	
Never do sports	583 (23.1)	475 (25.2)	0.128
Sport (h/week)	3.00 [2.00, 4.50]	2.50 [1.50, 4.00]	0.001
Comorbidities^b^			
Coronary heart disease	62 (2.4)	63 (2.4)	1.000
Heart faillure	51 (1.9)	48 (1.8)	0.826
Hypertension	732 (27.9)	633 (24.2)	0.002
Diabetes mellitus	90 (3.4)	116 (4.4)	0.074
Chronic lung disease	330 (12.6)	408 (15.6)	0.002
Chronic kidney disease	233 (8.9)	240 (9.3)	0.709
Cancer	305 (12.0)	243 (9.3)	0.002
Predictors Sarcopenia			
Skeletal muscle mass, kg	29.50 [22.70, 33.42]	27.20 [21.00, 32.60]	< 0.001
Hand grip strength, right, kg	36.95 [28.96, 46.18]	36.27 [28.69, 46.24]	0.531
Hand grip strength, left, kg	34.63 [27.17, 43.90]	34.17 [26.97, 44.38]	0.889
Timed up and go, s	6.00 [5.00, 7.00]	6.00 [6.00, 7.00]	0.317
Sarcopenia			
Prevalence Sarcopenia, n (%)	16 (3.2)	57 (3.6)	0.750

Continuous variables are presented as median and interquartile range, categorical variables as absolute numbers and percentages. Comparisons between groups were performed using Kruskal–Wallis test for continuous variables and Chi-square test for categorical variables.

^a^ Educational status defined by school and vocational qualifications according to International Standard Classification of Education (ISCED).

^b^ Self-reported.

Abbreviations: BMI = Body Mass Index.

### Musculoskeletal function in participants since begin of SARS-CoV-2 pandemic versus matched controls

The mean skeletal muscle mass was 29.50 kg (IQR: 22.70–33.42) in the pre-pandemic group and 27.20 kg (IQR: 21.00–32.60) in the post-pandemic group. The prevalence of sarcopenia was low (pre-pandemic group 3.2%, post-pandemic group 3.6%), which was most likely explained by the comparatively young age of the study population. However, the skeletal muscle mass showed statistically significant differences (*p* < 0.001) between the two groups, with reduced skeletal muscle mass observed in the post-pandemic group. In linear mixed regression analysis adjusted for body surface area, SARS-CoV-2 pandemic was associated with lower skeletal muscle mass [regression coefficient -0.337 (adjusted CI − 0.663, − 0.011), adjusted *p* = 0.038]. In the sensitivity analysis with additional adjustment for comorbidities, the results remained consistent with the main findings (Supplemental Table 1). When physical activity was included as an additional covariate, the associations with the pandemic variables were no longer statistically significant, suggesting that physical activity is likely a consequence of the pandemic and not an independent covariate (Supplemental Table 2).

**Table 2:** Baseline and functional characteristics: post-infection cohort vs. matched controls.

	Post-infection group (n = 428)	Non-infected matched controls (n = 1712)	*p*-value
Demographics			
Age, years	55.00 [51.00, 60.00]	55.00 [51.00, 60.00]	0.893
Sex, female	228 (53.3)	933 (54.5)	0.688
Education^a^			0.996
Low	7 (1.6)	27 (1.6)	
Medium	165 (38.6)	661 (38.6)	
High	256 (59.8)	1024 (59.8)	
Anthropometry			
BMI, kg/m^2^	25.91 [23.29, 28.84]	25.59 [23.14, 28.71]	0.370
Height, cm	174.75 [168.30, 181.55]	172.15 [165.67, 179.30]	< 0.001
Weight, kg	79.40 [70.30, 90.80]	76.95 [66.85, 87.70]	0.001
Body surface area (DuBois)	1.96 [1.80, 2.12]	1.90 [1.76, 2.05]	< 0.001
Relative fat mass, %	32.15 [27.25, 38.00]	32.00 [26.00, 38.16]	0.327
Lifestyle			
Status of employment			0.039
Full-time employed	238 (56.5)	907 (55.3)	
Part-time employed	101 (24.0)	328 (20.0)	
Marginal employed	24 (5.7)	79 (4.8)	
Not employed	18 (4.3)	112 (6.8)	
Pension	40 (9.5)	214 (13.0)	
Never do sports	57 (16.8)	344 (21.2)	0.084
Sport (h/week)	3.00 [2.00, 4.50]	3.00 [2.00, 4.50]	0.972
Comorbidities^b^			
Coronary heart disease	13 (3.1)	40 (2.4)	0.514
Heart faillure	8 (1.9)	36 (2.1)	0.907
Hypertension	101 (24.2)	475 (28.2)	0.117
Diabetes mellitus	14 (3.3)	65 (3.8)	0.719
Chronic lung disease	52 (12.4)	229 (13.6)	0.545
Chronic kidney disease	48 (11.4)	156 (9.3)	0.229
Cancer	41 (9.6)	233 (14.3)	0.014
SARS-CoV-2			
Self-reported disease severity at the time of infection			
Asymptomatic	13 (3.1)		
Mild	246 (58.6)		
Moderate, no need for hospitalization	131 (31.2)		
Moderate, need for hospitalization	30 (7.1)		
Predictors Sarcopenia			
Skeletal muscle mass, kg	26.75 [21.37, 31.83]	24.55 [20.56, 30.71]	0.021
Hand grip strength, right	35.13 [28.36, 47.15]	34.40 [28.20, 44.17]	0.076
Hand grip strength, left	33.88 [26.43, 44.59]	32.27 [26.13, 42.00]	0.142
Timed up and go, s	6.00 [6.00, 7.00]	6.00 [6.00, 8.00]	0.780
Sarcopenia			
Prevalence Sarcopenia, n (%)	4 (1.5)	9 (3.8)	0.177

Continuous variables are presented as median and interquartile range, categorical variables as absolute numbers and percentages.

Comparisons between groups were performed using Kruskal–Wallis test for continuous variables and Chi-square test for categorical variables.

^a^ Educational status defined by school and vocational qualifications according to International Standard Classification of Education (ISCED).

^b^ Self-reported.

Abbreviations: BMI = Body Mass Index.

**Fig. 2:**
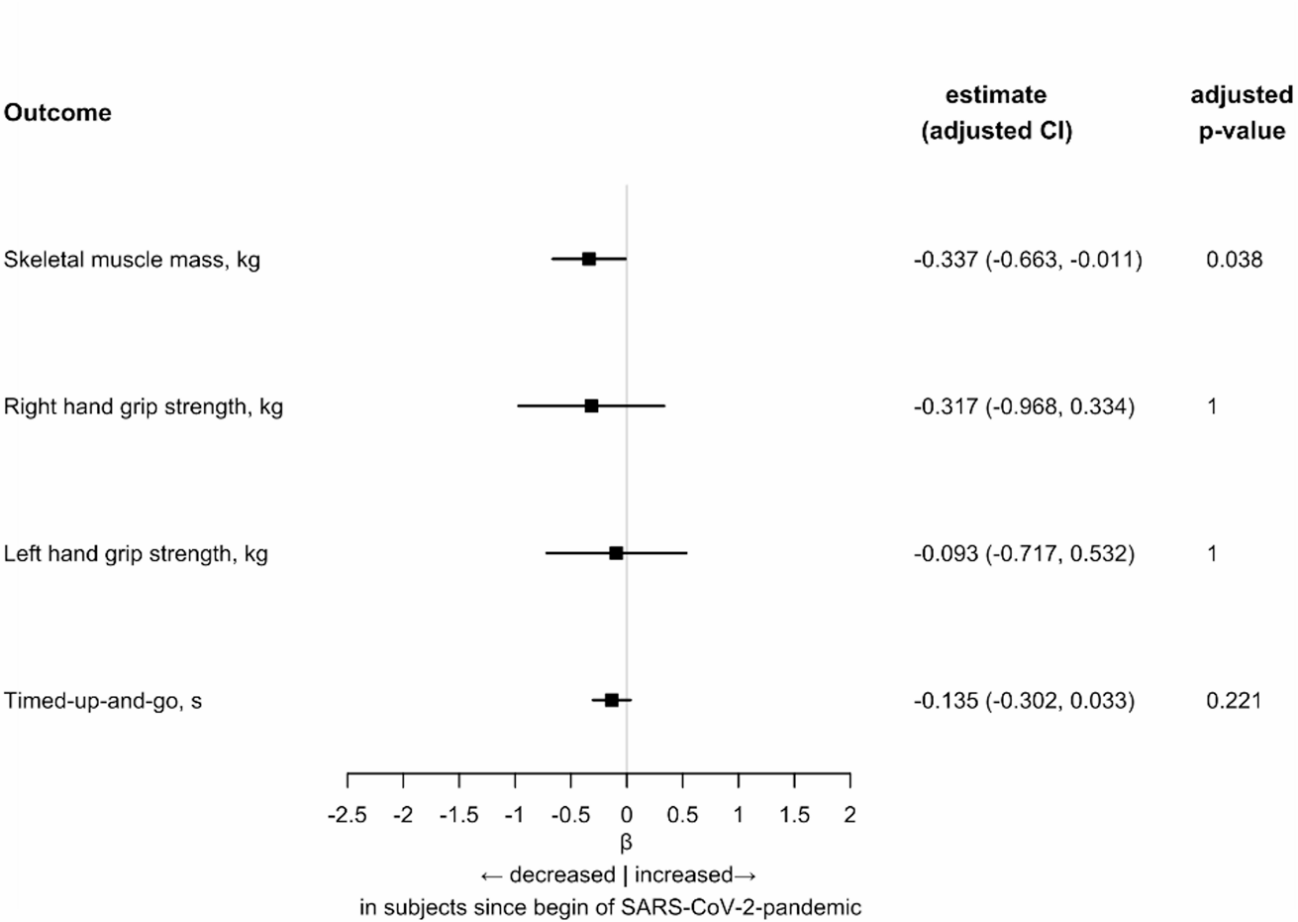
Forest plot showing the relationship between SARS-CoV-2 pandemic and musculoskeletal outcomes. Regression estimates for participants since begin of SARS-CoV-2 pandemic versus matched controls. Regression estimates are presented as beta and 95% confidence interval. Adjustment was performed for Body surface area (DuBois) and matching cluster was used as random intercept. *p*-values were adjusted for multiple testing with Bonferroni correction.

The mean handgrip strength was 36.95 kg (IQR: 28.96–46.18) on the right side and 34.63 kg (IQR: 27.17–43.90) on the left side in the pre-pandemic group, while in the post-pandemic group it was 36.27 kg (IQR: 28.69–46.24) on the right side and 34.17 kg (IQR: 26.97–44.38) on the left side. No statistically significant differences were observed between the two groups for the handgrip strength. The mean timed up and go test results were 6.00 s (IQR: 5.00–7.00) in the pre-pandemic group and 6.00 s (IQR: 6.00–7.00) in the post-pandemic group, with no statistically significant differences between the two groups (*p* = 0.317). Furthermore, linear mixed regression analysis adjusted for body surface area did not show any significant differences in hand grip strength or timed up and go between the pre-pandemic and post-pandemic groups (Fig. 2, Supplemental Table 3).

### Impact of SARS-CoV-2 infection on musculoskeletal parameters

This matched cohort analysis included a total of 2140 participants, with 428 participants who suffered a SARS-CoV-2 infection (infection group) and 1712 matched controls (non-infection group). The mean age of the participants was 55.0 years (IQR: 51.0–60.0) in both groups. Gender distribution displayed comparability, with females accounting for a significant portion in both the infection and non-infection groups. In the infection group, disease severity included mild (58.6%), moderate without hospitalization (31.2%), moderate with hospitalization (7.1%), and asymptomatic cases (3.1%). The severity of the disease was determined by a subjective self-assessment by the study participants, who classified their disease into one of the above categories. However, the temporal interval between the onset of infection and subsequent assessment at the study center was not included into the evaluation due to the inherently subjective nature of this self-reported data. Regarding education, both groups predominantly consisted of participants with medium and high education levels. Employment distribution was comparable, with variations in the proportion of employment types. In terms of physical attributes, the two groups differed in several aspects. The infection group had slightly higher mean values for BMI, height, weight and body surface area than the non-infection group. In particular, height, weight (*p* = 0.001) and body surface area (*p* < 0.001) showed statistically significant differences between the groups, with no significant difference in BMI. The mean relative fat mass was 32.15% (IQR: 27.25–38.00) in the infection group and 32.00% (IQR: 26.00–38.16) in the non-infection group, with no statistical significance. In terms of comorbidities, both the infection and non-infection groups had different rates of conditions such as coronary heart disease, heart failure, hypertension, diabetes mellitus, chronic lung disease, chronic kidney disease, with no significant differences. The prevalence of cancer (*p* = 0.014) was higher in the non-infection group. Regarding physical activity, participants from both the infection and non-infection groups reported their engagement in regular exercise. Notably, no significant difference was observed in the average number of hours spent on physical activity per week between the two groups. For detailed baseline characteristics, refer to Table 2.

### Musculoskeletal function in participants with a SARS-CoV-2 infection versus matched controls

The mean skeletal muscle mass was 26.75 kg (IQR: 21.37–31.83) in the infection group and 24.55 kg (IQR: 20.56–30.71) in the non-infection group. The prevalence of sarcopenia was low (infection group 1.5%, non-infection group 3.8%), which was again most likely explained by the comparatively young age of the study participants.

Only skeletal muscle mass (*p* = 0.021) showed statistically significant differences between the two groups, with reduced skeletal muscle mass observed in the non-infection group.

**Fig. 3:**
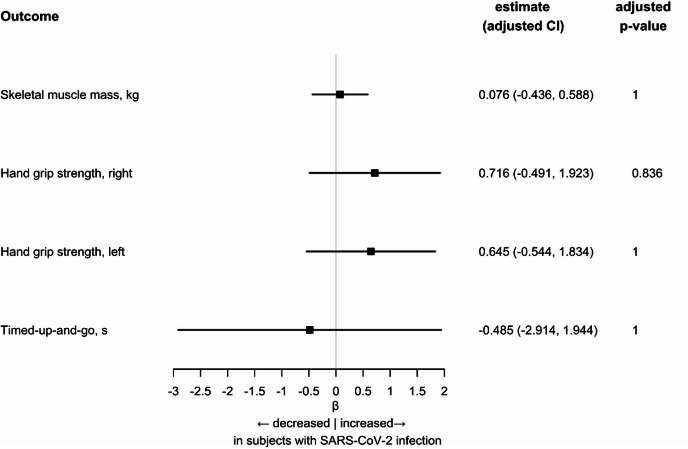
Forest plot showing the relationship between SARS-CoV-2 infection and musculoskeletal outcomes. Regression estimates for participants after SARS-CoV-2 infection versus non-infected matched controls. Regression estimates are presented as beta and 95% confidence interval. Adjustment was performed for Body surface area (DuBois) and matching cluster was used as random intercept. *p*-values were adjusted for multiple testing with Bonferroni correction.

The mean handgrip strength was 35.13 kg (IQR: 28.36–47.15) on the right side and 33.88 kg (IQR: 26.43–44.59) on the left side in infection group, while in the non-infection group it was 34.40 kg (IQR: 28.20–44.17) on the right side and 32.27 kg (IQR: 26.13–42.00) on the left side. No statistically significant differences were observed between the two groups for the handgrip strength. The mean timed up and go test results were 6.00 s (IQR: 6.00–7.00) in the infection group and 6.00 s (IQR: 6.00–8.00) in the non-infection group, with no statistically significant differences between the two groups (*p* = 0.780). Despite the Kruskal–Wallis test indicating a significant difference in skeletal muscle mass, the linear mixed regression analysis adjusted for body surface area did not show any significant differences in skeletal muscle mass, hand grip strength or timed up and go between the infection and non-infection groups (Fig. 3, Supplemental Table 4). Sensitivity analyses adjusting for comorbidities supported the main findings (Supplemental Table 5). Inclusion of physical activity attenuated the effects, probably reflecting reduced activity as a result of the pandemic (Supplemental Table 6).

## Discussion

This prospective study aimed to explore the impact of the global SARS-CoV-2 pandemic on indicators of musculoskeletal health. The SARS-CoV-2 pandemic was approached from multiple angles, using data obtained from both SARS-CoV-2 non-infected and SARS-CoV-2 infected individuals. With this approach, the study first aimed to examine how the pandemic and the resulting changes in daily life could affect the musculoskeletal system. Secondly, it aimed to investigate the potential effects of a SARS-CoV-2 infection on established indicators of musculoskeletal health. The data of this trial show no association between SARS-CoV-2 infection and musculoskeletal changes in the cohort studied, overall indicating that SARS-CoV-2 infections may not be a significantly associated with major deterioration of musculoskeletal function. The findings further suggest that lifestyle changes in daily routines and physical activities associated during the pandemic might negatively affect specific parameters of musculoskeletal health.

To assess the influence of the SARS-CoV-2 pandemic on the musculoskeletal system, two cohorts of non-infected participants were compared: one before the pandemic and one during the pandemic at one single time point. In this context, the baseline characteristics of the participants, including sociodemographic information, comorbidities, and anthropometric data, were comparable and similar in both groups. This suggests that the groups were adequately matched. The study revealed that the average skeletal muscle mass, a measure of muscle quantity, was significantly lower in the post-pandemic group as compared to the pre-pandemic group. The observed decrease in skeletal muscle mass within the post-pandemic group might be attributed to a combination of factors influenced by the pandemic-induced lifestyle changes. Lockdowns, social distancing measures, and increased time spent indoors could have contributed to a reduction in physical activity^19–21^. This hypothesis is supported by the fact that participants in the post-pandemic group reported a statistically significant lower average number of hours of physical activity per week. Reduced physical activity has been associated with muscle atrophy^22,23^ and a shift in body composition towards a higher fat content^24^. Additionally, other factors, such as changes in dietary habits, might have played a role^25,26^. Tzeng et al. examined lifestyle behaviors in older adults independently of the SARS-CoV-2 pandemic to assess their risk of musculoskeletal impairment. They demonstrated that an unbalanced diet and reduced physical activity were associated with an increased risk of functional and structural muscle loss in this age group^27^. Batista et al. and Saraiva et al. specifically examined the impact of the SARS-CoV-2 on the mobility of older adults and the associated risk of musculoskeletal impairment. Their findings indicated an increased prevalence of impairments in muscle mass and function in this group. In particular, reduced life space mobility was a significant contributor to muscle loss in older adults during the SARS-CoV-2 pandemic^28,29^. Similarly, the psychological aspects associated with the pandemic could have contributed. Stress-induced changes in hormone levels may influence alterations in body composition. Moreover, heightened stress levels can potentially lead to the adoption of unhealthy eating patterns and consequent weight gain^30,31^. Other research has shown similar results: Hasegawa et al. investigated the impact of SARS-CoV-2 pandemic measures on muscle mass in older type 2 diabetes patients. Their analysis of bioelectrical impedance data from 56 patients, with an average age of 75.2 years, revealed a significant decline in muscle mass during the SARS-CoV-2 pandemic. They propose that the restrictions on physical activity may have contributed to the muscle loss in these older patients^32^. Kirwan et al. conducted a comprehensive review of the causes of muscle mass loss and compared them with data showing how the SARS-CoV-2 lockdowns affected physical activity, diet, sleep and stress. Their findings suggest that the lockdowns, which lowered physical activity levels and altered dietary habits, may increase the risk of muscle loss, especially in older individuals^7^. In a similar study, Woods et al. discussed the further effects of the SARS-CoV-2 pandemic on health and daily life. The study indicates that the reduced physical activity caused by quarantine and social distancing may have a negative impact on our muscles and general physical health^33^.

However, it is important to acknowledge that impairments in mobility and musculoskeletal function in this study may not entirely capture the effects of social isolation on functional abilities. Although a reduction in skeletal muscle mass was observed, the evaluation of muscle strength via handgrip and physical performance assessed through the timed up and go test showed no significant differences between the groups. The absence of substantial differences in these metrics suggests a relatively restrained effect of the pandemic on these particular parameters in this particular study cohort at a single time measurement. However, in the post-pandemic cohort, the average age with a median age is 54.0 years in both groups. Since reduced musculoskeletal function is a syndrome associated with aging^34^, it might be plausible that the observed effects primarily represent precursors, potentially indicative of a higher propensity for the development of progressive muscle mass and function loss in individuals over time. Caution is needed when interpreting this hypothesis, but it could provide useful insights for future research, particularly in studies targeting the pandemic effects especially on older populations.

In order to evaluate the impact of a SARS-CoV-2 infection on the musculoskeletal system, study participants who had experienced a SARS-CoV-2 infection were matched with a non-infected cohort. While the baseline characteristics were effectively matched, differences in physical attributes such as BMI, height, and weight surfaced between the infection and pandemic cohorts were found. Participants in the infection group had significant differences in height and weight, being significantly taller with a median of 2.60 cm and heavier with a median of 2.45 kg. However, physical activity levels were similar in both groups, with participants from each group reporting engagement in regular physical exercise. Furthermore, the average weekly duration spent on physical activities demonstrated consistency across the two groups. The similarity in activity engagement suggests that participants in both cohorts likely maintained comparable levels of physical activity. This, in turn, might have played a role in preserving musculoskeletal health, even in the context of acute infections and subsequent periods. Despite the initial finding of reduced skeletal muscle mass in the non-infected group, when accounting for body surface area through regression analysis, the differences in skeletal muscle mass, hand grip strength, and timed up and go test between the infection and non-infection groups were not statistically significant. This suggests that the initial observed differences may have been influenced by variations in body size and are not in association with a SARS-CoV-2 infection. Similar findings were reported by Liu et al. They investigated the association between a SARS-CoV-2 infection and reduced muscle function and mass using data from the UK Biobank. To identify potential associations, they performed various Mendelian randomization analyses to test for causality. However, their results did not provide strong evidence of a direct causal relationship between a SARS-CoV-2 infection and reduced muscle function and mass. While the study investigated the direct causal relationship between a SARS-CoV-2 infection and the loss of muscle mass and function, the results suggest a possible indirect interaction^35^. However, other studies analyzing the effect of a SARS-CoV-2 infection have shown that particularly severe cases of an infection, characterized by prolonged disease, can lead to muscle atrophy and dysfunction^36,37^. Notably, our study did not include this specific group of patients with severe disease progression.

In terms of progressive and generalized loss of muscle mass and function, the current data show that the prevalence was relatively low in both the infection and non-infection groups. This study contributes to the understanding of the relationship between a SARS-CoV-2 infection and the musculoskeletal system by highlighting that, in the context of mild to moderate infections and short durations, the virus does not appear to lead to substantial musculoskeletal deterioration or a significantly increased risk of progressive and generalized loss of muscle mass and function. However, it’s important to note that the study’s findings are based on specific parameters and characteristics of the participants, and that the impact of SARS-CoV-2 infection on musculoskeletal health may still vary depending on factors such as disease severity, duration, and individual health conditions. Furthermore, it is important to note that the generalizability of these findings is limited by the relatively young and healthy nature of our study cohort, which may not reflect more vulnerable populations at higher risk or severe COVID-19 outcomes.

### Limitations

There are several limitations to this study. First, the average age of the participants, ranging from early to mid-50 s, is comparably young in the context of progressive and generalized loss of muscle mass and function, which primarily effects older patients. This demographic characteristic is also reflected in the relatively low prevalence of sarcopenia observed in this study. To address this limitation, future studies should specifically focus on elderly populations to investigate the impact of SARS-CoV-2 infection, pandemic-related lifestyle changes, and similar public health crises on musculoskeletal health. Another important limitation is the potential for selection bias, as the study cohort predominantly consisted of relatively healthy and mobile individuals. The demanding nature of the 7-h study visit likely discouraged participation by those with significant health impairments or physical limitations. Consequently, the resulting cohort may not adequately represent more vulnerable or clinically relevant populations^38^. In addition, the available data only include assessments of participants with asymptomatic to mild infection. The dataset lacks information on more severe infection courses and their impact on musculoskeletal health. Furthermore, there is no data available on virus variants or vaccination status of the post-pandemic cohorts. Finally, the use of BIA to assess muscle mass can be considered a limitation, as this method has certain constraints compared to gold-standard techniques such as DXA^39^. However, BIA is recommended by current guidelines for the assessment of body composition and muscle-related conditions, including sarcopenia, given that all participants were measured under standardized conditions, the results remain internally comparable and valid for group comparisons.

## Conclusion

Although it is known that the SARS-CoV-2 pandemic affects organ systems in various ways, this study shows that there is no association between SARS-CoV-2 infection and changes in the musculoskeletal outcome parameters assessed in this study. The absence of significant differences in key parameters such as muscle strength, quality and performance between the infection and non-infection groups emphasizes that a SARS-CoV-2 infection may not be a major driver of musculoskeletal changes. In fact, the predominant influence seems to be the changes in lifestyle and physical activity brought about by the pandemic itself. Together, this study supports the hypothesis that lifestyle factors, such as physical activity and daily routine alterations, have a greater impact on the musculoskeletal system than the temporary effects of a mild to moderate infection from SARS-CoV-2.

## Supplementary Information

Below is the link to the electronic supplementary material.

Supplementary Material 1

Supplementary Material 2

Supplementary Material 3

Supplementary Material 4

Supplementary Material 5

Supplementary Material 6

## Data Availability

All relevant data are presented in the figures and tables of the manuscript. Further data supporting the findings of this study are not publicly available due to sensitivity reasons but can be obtained from the corresponding author upon reasonable request.
