# Supplementary material for: Influence of the SARS-CoV-2 pandemic and infection on musculoskeletal function
Source: Sci Rep. 2025 Sep 12;15:32510. doi: 10.1038/s41598-025-17780-x (PMC12432240; doi:10.1038/s41598-025-17780-x)
Supplement: Supplementary file 1 — Supplementary Material 1 [file 41598_2025_17780_MOESM1_ESM.docx]

**Supplemental Table 1. Sensitivity analysis including comorbidities: pandemic cohort vs. matched controls**

|  | **n** | **beta (95%-CI)** | **p-Value** | **beta (adjusted CI)** | **p-Value (adjusted)** |
| --- | --- | --- | --- | --- | --- |
| **Predictors Sarcopenia** |  |  |  |  |  |
| Skeletal muscle mass, kg | 2822 | -0.343 (-0.583, -0.103) | 0.005 | -0.343 (-0.68, -0.007) | 0.043 |
| Right hand grip strength, kg | 3887 | -0.523 (-1.005, -0.041) | 0.034 | -0.523 (-1.196, 0.15) | 0.268 |
| Left hand grip strength, kg | 3888 | -0.26 (-0.718, 0.197) | 0.265 | -0.26 (-0.899, 0.379) | 1 |
| Timed up and go, s | 2158 | -0.13 (-0.25, -0.009) | 0.035 | -0.13 (-0.298, 0.039) | 0.283 |

Regression estimates for probands during the SARS-CoV-2 pandemic vs. matched controls (adjusted for body surface area, hypertension, chronic lung disease and cancer). Regression estimates are presented as beta and 95% confidence interval.
