# Supplementary material for: Influence of the SARS-CoV-2 pandemic and infection on musculoskeletal function
Source: Sci Rep. 2025 Sep 12;15:32510. doi: 10.1038/s41598-025-17780-x (PMC12432240; doi:10.1038/s41598-025-17780-x)
Supplement: Supplementary file 2 — Supplementary Material 2 [file 41598_2025_17780_MOESM2_ESM.docx]

**Supplemental Table 2. Sensitivity analysis including physical activity: pandemic cohort vs. matched controls**

|  | **n** | **beta (95%-CI)** | **p-Value** | **beta (adjusted CI)** | **p-Value (adjusted)** |
| --- | --- | --- | --- | --- | --- |
| **Predictors Sarcopenia** |  |  |  |  |  |
| Skeletal muscle mass, kg | 1647 | -0.271 (-0.563, 0.021) | 0.07 | -0.271 (-0.683, 0.141) | 0.563 |
| Right hand grip strength, kg | 2600 | -0.473 (-1.077, 0.131) | 0.125 | -0.473 (-1.319, 0.372) | 1 |
| Left hand grip strength, kg | 2601 | -0.05 (-0.633, 0.533) | 0.866 | -0.05 (-0.866, 0.766) | 1 |
| Timed up and go, s | 1455 | -0.214 (-0.363, -0.066) | 0.005 | -0.214 (-0.423, -0.006) | 0.04 |

Regression estimates for probands during the SARS-CoV-2 pandemic vs. matched controls (adjusted for body surface area, sport (h/week)). Regression estimates are presented as beta and 95% confidence interval.
