# Supplementary material for: Influence of the SARS-CoV-2 pandemic and infection on musculoskeletal function
Source: Sci Rep. 2025 Sep 12;15:32510. doi: 10.1038/s41598-025-17780-x (PMC12432240; doi:10.1038/s41598-025-17780-x)
Supplement: Supplementary file 3 — Supplementary Material 3 [file 41598_2025_17780_MOESM3_ESM.docx]

**Supplemental Table 3. Regression results: pandemic cohort vs. matched controls**

|  | **β (95%-CI)** | **Nominal p-value** | **Adjusted CI^a^** | **Adjusted p-value^a^** |
| --- | --- | --- | --- | --- |
| **Predictors Sarcopenia** |  |  |  |  |
| Skeletal muscle mass, kg | -0.337 (-0.570, -0.104) | 0.005 | -0.337 (-0.663, -0.011) | 0.038 |
| Right hand grip strength, kg | -0.317 (-0.783, 0.149) | 0.182 | -0.317 (-0.968, 0.334) | 1.000 |
| Left hand grip strength, kg | -0.093 (-0.540, 0.354) | 0.685 | -0.093 (-0.717, 0.532) | 1.000 |
| Timed up and go, s | -0.135 (-0.255, -0.015) | 0.028 | -0.135 (-0.302, 0.033) | 0.221 |

Regression estimates for patients since begin of SARS-CoV-2 pandemic versus matched controls. Regression estimates are presented as beta and 95% confidence interval. Adjustment was performed for Body surface area (DuBois) and matching cluster was used as random interept.

^a^ adjusted for multiple testing with Bonferroni correction.
