# Supplementary material for: Influence of the SARS-CoV-2 pandemic and infection on musculoskeletal function
Source: Sci Rep. 2025 Sep 12;15:32510. doi: 10.1038/s41598-025-17780-x (PMC12432240; doi:10.1038/s41598-025-17780-x)
Supplement: Supplementary file 4 — Supplementary Material 4 [file 41598_2025_17780_MOESM4_ESM.docx]

**Supplemental Table 4. Regression results: post-infection cohort vs. matched controls**

|  | β (95%-CI) | Nominal p-value | Adjusted CI^a^ | Adjusted p-value^a^ |
| --- | --- | --- | --- | --- |
| **Predictors Sarcopenia** |  |  |  |  |
| Skeletal muscle mass, kg | 0.076 (-0.288, 0.440) | 0.683 | 0.076 (-0.436, 0.588) | 1.000 |
| Right hand grip strength, kg | 0.716 (-0.148, 1.580) | 0.105 | 0.716 (-0.491, 1.923) | 0.836 |
| Left hand grip strength, kg | 0.645 (-0.206, 1.496) | 0.138 | 0.645 (-0.544, 1.834) | 1.000 |
| Timed up and go, s | -0.485 (-2.212, 1.242) | 0.582 | -0.485 (-2.914, 1.944) | 1.000 |

Regression estimates for patients after SARS-CoV-2 infection versus matched controls. Regression estimates are presented as beta and 95% confidence interval. Adjustment was performed for Body surface area (DuBois) and matching cluster was used as random interept.

^a^ adjusted for multiple testing with Bonferroni correction.
