# Supplementary material for: Influence of the SARS-CoV-2 pandemic and infection on musculoskeletal function
Source: Sci Rep. 2025 Sep 12;15:32510. doi: 10.1038/s41598-025-17780-x (PMC12432240; doi:10.1038/s41598-025-17780-x)
Supplement: Supplementary file 5 — Supplementary Material 5 [file 41598_2025_17780_MOESM5_ESM.docx]

**Supplemental Table 5. Sensitivity analysis including comorbidities: post-infection cohort vs. matched controls**

|  | **n** | **beta (95%-CI)** | **p-Value** | **beta (adjusted CI)** | **p-Value (adjusted)** |
| --- | --- | --- | --- | --- | --- |
| **Predictors Sarcopenia** |  |  |  |  |  |
| Skeletal muscle mass, kg | 624 | 0.039 (-0.345, 0.423) | 0.842 | 0.039 (-0.501, 0.579) | 1 |
| Right hand grip strength, kg | 1676 | 0.483 (-0.419, 1.386) | 0.294 | 0.483 (-0.777, 1.744) | 1 |
| Left hand grip strength, kg | 1675 | 0.339 (-0.547, 1.225) | 0.454 | 0.339 (-0.899, 1.577) | 1 |
| Timed up and go, s | 565 | -0.438 (-2.142, 1.267) | 0.615 | -0.438 (-2.838, 1.963) | 1 |

Regression estimates for post-infection vs. matched controls (adjusted for body surface area, hypertension, chronic lung disease and cancer). Regression estimates are presented as beta and 95% confidence interval.
