# Supplementary material for: Influence of the SARS-CoV-2 pandemic and infection on musculoskeletal function
Source: Sci Rep. 2025 Sep 12;15:32510. doi: 10.1038/s41598-025-17780-x (PMC12432240; doi:10.1038/s41598-025-17780-x)
Supplement: Supplementary file 6 — Supplementary Material 6 [file 41598_2025_17780_MOESM6_ESM.docx]

**Supplemental Table 6. Sensitivity analysis including physical activity: post-infection cohort vs. matched controls**

|  | **n** | **beta (95%-CI)** | **p-Value** | **beta (adjusted CI)** | **p-Value (adjusted)** |
| --- | --- | --- | --- | --- | --- |
| **Predictors Sarcopenia** |  |  |  |  |  |
| Skeletal muscle mass, kg | 453 | 0.046 (-0.396, 0.489) | 0.837 | 0.046 (-0.581, 0.674) | 1 |
| Right hand grip strength, kg | 1227 | 0.231 (-0.814, 1.275) | 0.666 | 0.231 (-1.231, 1.692) | 1 |
| Left hand grip strength, kg | 1226 | 0.209 (-0.83, 1.247) | 0.694 | 0.209 (-1.245, 1.662) | 1 |
| Timed up and go, s | 413 | -0.017 (-1.919, 1.886) | 0.986 | -0.017 (-2.713, 2.68) | 1 |

Regression estimates for post-infection vs. matched controls (adjusted for body surface area, sport (h/week)). Regression estimates are presented as beta and 95% confidence interval.
